# Progress in Photo-Therapy

**Published:** 1903-05-30

**Authors:** 


### Editor's Letter-Box.

[Our Correspondents are reminded that prolixity is a great bar to publication, and that brevity of style and conciseness of statement greatly facilitate early insertion.]

### PROGRESS IN PHOTO-THERAPY.

Mr. Stephen Mayou points out that the first case of trachoma cured by *x*-rays by himself was published in *Trans. Ophth. Soc.*, 1902, some six months before Messrs. Stephenson and Walsh published their case. See also *Arch. Roentgen Society*, December, 1901, where 15 cases are quoted.

